# Magnitude and associated factors of intra-operative hypothermia among pediatric patients undergoing elective surgery: A multi-center cross-sectional study

**DOI:** 10.1016/j.amsu.2022.103338

**Published:** 2022-02-08

**Authors:** Getachew Mekete, Geresu Gebeyehu, Sulaiman Jemal, Yewlsew Fentie

**Affiliations:** a Department of Anesthesia, College of Health Sciences, School of Medicine, Debre Tabor University, Debre Tabor, Ethiopia; b Department of Anesthesia Addis Ababa University School of Medicine, Addis Ababa, Ethiopia

**Keywords:** Hypothermia, Intra-operative, Magnitude, Risk factors, Ethiopia

## Abstract

**Background:**

Hypothermia is a core body temperature of less than 36 c^0^ that could increase perioperative morbidity & mortality of pediatric patients operated under general anesthesia. This study aimed to assess the magnitude and associated factors of intra-operative hypothermia among pediatric patients undergoing elective surgery.

**Method:**

Institutional based cross-sectional study was conducted on 339 pediatric patients undergoing elective surgery in referral hospitals of Addis Ababa, Ethiopia. Data were collected using a structured questionnaire. Descriptive statics were expressed in percentage and presented with tables. Both Bivariable and multivariable logistic analysis was done to identify factors associated with intra-operative hypothermia among pediatric patients undergoing elective surgery. P < 0.05 with 95% CI was set as statistical significance.

**Result:**

The magnitude of intra-operative hypothermia in this study was 39.8 [95% CI= (34.5–45.1)]. Multivariable logistic analyses showed that ambient temperature less than 26c^0^ [AOR = 6; 95%CI=(2.859–13.23)], volume of fluid administered greater than half a liter [AOR = 3.6; 95%CI=(1.83–7.23)], Patients who were received un-warm fluid [AOR = 2.2; 95%CI=(1.28–4.04)] and duration of surgery and anesthesia greater than 120 min [AOR = 3.4; 95%CI=(1.29–8.79)] and [AOR = 3.8; 95%CI=(1.25–11.53)] respectively were factors significantly associated with intra-operative hypothermia.

**Conclusion:**

The magnitude of intra-operative hypothermia in this study was high. So, adherence must be done in the prevention of intraoperative hypothermia by health professionals such as increasing operation room temperature, warming administered fluid, optimal fluid administration, and reduction of the duration of surgery and anesthesia.

## Highlights

• Intraoperative hypothermia causes unnecessary complications in pediatrics patients.
• Incidence of intraoperative hypothermia in pediatrics patients was high.
• Prevention of hypothermia is vital in reduction of the incidence intraoperative hypothermia.

## 1 Introduction

Hypothermia is a core body temperature of less than 36c^0^ frequently encountered in pediatric patients operated under general anesthesia [1,2]. Maintaining normothermia is vital for patient comfort and the prevention of complications caused by hypothermia [[3], [4], [5]].

Intraoperative hypothermia during surgery and anesthesia affects more than 70% of patients during surgery and anesthesia. The incidence of intraoperative hypothermia in pediatric patients is higher than preoperative and postoperative hypothermia [[6], [7], [8]].

Intraoperative hypothermia occurs with four mechanisms, radiation (50–70%), convection (15–25%), evaporation (5–15%), and conduction (3–5%) [9,10].

Pediatric patients are at high risk of hypothermia due to easy heat loss caused by higher surface area to volume ratio, immature hypothalamic thermoregulatory capacity, and less insulating subcutaneous tissue [[11], [12], [13]]. Also, impairment of the non-shivering thermogenesis mechanism of brown fat for heat production during general anesthesia plays a great role in hypothermia [[14], [15], [16], [17]].

Anesthetic-induced impaired thermoregulation center, inhibition of normal response to heat loss, cooling effect of cold anesthetic gases, and heat loss to the environment and cold operation room [[18], [19], [20], [21]], causes complications of coagulopathy, delaying of drug metabolism, and prolonged recovery [[18], [19], [20], [21]].

Despite evidence is limited regarding the magnitude and associated factors of intraoperative hypothermia in the pediatric age group in the study areas. So, this study is important to prevent and manage intraoperative hypothermia in pediatrics patients undergoing general surgery.

## 2 Methods and materials

This cross-sectional study was conducted in the capital city, Addis Ababa, Ethiopia in public referral hospitals. The altitude of the city ranges from 2200 to 3000 m above sea level with an average temperature of 22.8 °C. According to the 2021 census, about 5005524 people live in the city. There are 40 hospitals, 29 health Centers, 122 health stations, 37 health posts, and 382 private clinics in Addis Ababa. Out of 13 public hospitals, seven of them provide pediatric surgery services. While this study was conducted in four referral hospitals: Black Lion specialized Hospital, Zewditu Memorial Hospital, St. Paul Millennium hospital, and Minilik II Hospital from February January 2021 up to April, 30/2021. This study is registered at researchregistry7388. Also, it is reported according to STROCSS criteria [22].

### 2.1 Inclusion and exclusion criteria

Pediatric patients who were less than 18 years old were included in the study. While procedures with anesthesia duration less than 30 min, patients who have sustained one or more traumatic injuries, Patients with body temperature greater than 38^0c^ and less than 36^0c^, patients who underwent open-heart surgery, and a patient who refused were excluded from the study.

### 2.2 Sample size

The sample size was determined by using single proportion formula considering the following assumptions: 95% confidence interval (Z _α/2_), 5% margin of error(d), 30% incidence of intraoperative hypothermia(P) [23], and considering 5% non-response rate, finally, 339 sample size was obtained.

### 2.3 Sampling technique and procedure

A systematic random sampling technique was used to get the study participants. The total numbers of pediatrics patients included in the study were propotionated depending on the number of three-month elective surgeries done in each referral Hospital, using a formula nj=(n/N)Nj. Also, study participants from each hospital were selected using the systematic random sampling technique on daily operation schedule lists. Depending on situational analysis of 3 months, an average of 500 patients were operated. Then sampling interval (K) was determined using the formula: K=N/n; 500/339 = 1.47 ≈ 2 Where, n = total sample size, N = population per 3 months. Taking sampling interval two, the first study participant (random start) was selected using the lottery method from the daily surgery list.

### 2.4 Data collection instrument and procedures

Data was collected by using an English version structured questionnaire taken from studies. The temperature of patients was measured intraoperative using a digital axillary thermometer with an accuracy of ± 0.1 c^o^ by placing the probe in the armpit close to the axillary artery by tightly adducting the arm. Intraoperative body temperature was taken after surgery before the extubation of the patient. The ambient temperature was also recorded after the patient enters in operation room by using a digital room thermometer after providing training for data collectors and having verbal informed consent taken from their parent's data was collected using a questionnaire. The data was collected by four trained anesthetists; one data collector for each hospital after informed consent was taken from parents.

### 2.5 Data quality assurance

After training was given to data collectors, data were collected and properly filled in the prepared format. The supervision was made throughout the data collection period to make sure the accuracy, clarity, and consistency of the collected data. A pre-test was done on 5% of the sample size from Yikatit 12 referral hospital.

### 2.6 Ethical consideration

The ethical clearance to conduct the study was obtained from 10.13039/501100007941Addis Ababa University institutional review board and an Official support letter was given from referral hospitals of Addis Ababa as well informed written consent was secured from each study participant's family. Confidentiality was assured throughout the research.

### 2.7 Data entry, analysis, and interpretation

The collected data were coded, entered, and analyzed using SPSS version 26. Descriptive statistics were carried out and expressed in text, tables, and figures, and both bi-variable and multivariable logistic regression analyses were used to identify factors associated with the intraoperative hypothermia of patients undergoing elective surgery. Variables with a p-value of less than <0.2 in the bivariable logistic analysis were fitted into a multivariable logistic regression analysis. Both crude odds ratio (COR) in bivariable logistic regression and adjusted odds ratio (AOR) in multivariable logistic regression with the corresponding 95% confidence interval were calculated to show the strength of association. In multivariable logistic regression analysis, variables with a p-value <0.05 were considered statistically significant.

### 2.8 Operational definition

Normothermia: A core temperature range of 36 °C–38 °C [23,24].

Hyperthermia: A core temperature greater than 39 °C [23,24].

Hypothermia: A core temperature less than 36 °C [23,24].

Intra-operative hypothermia is defined as a temperature of patients less than 36 °C that lasted for at least 10 min [11,25].

Pediatric patient: a patient whose age is less than 18 years old [26].

## 3 Results

### 3.1 Socio-demographic characteristics of study participants

A sample of 339 study participants was involved in this study with a full response rate. The majority of participants in this study were males 207 (61.1%). The weight of study participants with Mean ± SD was 13.58 ± 8.4 kg.

The highest number of age groups were found between 1 and 12 months 112 (33%) and 220 (64.9%) of participants were ASA 1. About 85 (25.1%) pediatrics patients were with coexisting diseases (Table-1).

**Table: 1:** Socio-demographic characteristics of pediatrics patients (n = 339).

Variables	Category	Frequency	Percentage (%)
Age	Neonate	41	12.1
	Infant	112	33
	Toddler	104	30.7
	Children	69	20.4
	Adolescent	13	3.8
Sex	Male	207	61.1
	Female	132	38.9
Specific Medical Disease	CHD	30	35.7
	DM	19	22.6
	HTN	15	17.9
	Others	20	23.8
ASA States	ASAI	220	64.9
	ASAII	98	28.9
	ASAIII	21	6.2
Coexisting	Yes	85	25.1
	No	254	74.9

### 3.2 Anesthetic and surgical characteristics of study participants

Gastrointestinal surgery 82 (24.2%) was done in the majority of study participants while 237 (69.9%) were done under general anesthesia without any combined regional techniques. The Mean ± SD operation room temperature was 24.28 ± 2. The majority of patients were done with an operation room temperature of less than 26 c^o^, 247 (72.9%). Duration surgery and anesthesia were 126.6 ± 29.3 min and 139.7 ± 31.5 min respectively (Table-2).

**Table 2:** Intra-operative surgery and anesthesia-related factors of pediatrics patients (n = 339).

Variables	Category	Frequency	Percentage (%)
Operation room temperature	<26c^o^	252	74.3
	≥26c^o^	87	25.7
Surgery type	Orthopedic	32	9.4
	Neurosurgery	59	17.4
	ENT	50	14.7
	Urology	66	19.5
	Gastrointestinal	82	24.2
	General	27	80
	Thoracic	23	6.8
Anesthesia Type	General only	237	69.9
	Combined	102	30.1
Induction agent	Ketamine	30	8.8
	Thiopental	60	17.7
	Propofol	124	36.6
	Ketamine & Propofol	125	36.9
Relaxant agent	suxamethonium	69	20.4
	Vecuronium	13	3.8
	Pancuronium	4	1.2
	Suxamethonium & Vecronim	201	59.3
	suxamethonium & Pancuronium	52	15.3
Analgesic agent	Pethedine	136	40.1
	Morphine	119	35.1
	Tramadol	38	11.2
	Other	46	13.6
Volume of Fluid administered	≤500	182	53.7
	≥500 ml	157	46.3
Warmed fluid administer	yes	165	48.7
	No	174	51.3
Is blood transfused	Yes	48	14.2
	No	291	85.8
Duration of Surgery time	<120 min	138	40.7
	≥120 min	201	59.3
Duration of Anesthesia time	≤120 min	111	32.7
	≥120 min	228	67.3

### 3.3 Magnitude of intra-operative hypothermia

In this study, the magnitude of intra-operative hypothermia of pediatric patients undergoing elective surgery with general anesthesia was 39.8% [95% CI= (34.5–45.1)].

### 3.4 Factor associated with intra-operative hypothermia

The bivariable logistic analyses showed that Age, ASA PS, room temperature, un-warm fluid administered, the volume of fluid administered, duration of surgery, and anesthesia were factors associated with intra-operative hypothermia. Also, in multivariable logistic recreation neonates [AOR = 15; 95%CI=(2.256–101.8)], and infant age groups [AOR = 8.4; 95%CI=(1.548,45.86)] were more likely to develop intra-operative hypothermia than adolescents. Patients who have done surgery and anesthesia at an ambient temperature of less than 26c^0^were [AOR = 6; 95%CI=(2.859–13.231)] more likely to develop inter-operative hypothermia than those who underwent surgery with room temperature greater than 26c^o^.

Patients who received greater than half-liter were [AOR = 3.6; 95%CI=(1.83–7.23)] more likely to develop inter-operative hypothermia than patients who were received less than 500 ml. Also, unwarm fluid administration during intra-operatively was [AOR = 2.2; 95%CI=(1.28–4.05)] more likely to develop intra-operative hypothermia compared with received warm fluid. Duration of surgery More than a 120-min [AOR = 3.4; 95%CI=(1.297–8.797)] and duration of anesthesia More than a 120-min (AOR = 3.8; 95%CI=(1.25–11.53)] was more likely to develop intra-operative hypothermia than their counterparts (Table 3).

**Table 3:** Factor associated with pediatrics intra-operative Hypothermia (n = 339).

Variables	Intra-operative hypothermia		COR (95%CI)	AOR (95%CI)	P-value
	yes	NO			
**Age group**					
Neonate	24 (58.54%)	17 (41.46%)	4.7 (1.12,19.7)	15 (2.256,101.8)	0.005*
Infant	48 (42.85%)	64 (57.15%)	2.5 (0.665,9.58)	8.4 (1.548,45.86)	0.014*
Toddler	38 (36.5%)	66 (63.5%)	1.9 (0.49,7.4)	4.4 (0.84,23.148)	0.079
Children	22 (31.89%)	47 (68.11%)	1.5 (0.39,6.24)	2.3 (95%,	0.3
Adolescent	3 (23.1%)	10 (76.9%)	1	1	
**ASA PS**					
ASAI	72 (32.7%)	148 (67.3%)	1	1	
ASAII	53 (54.1%)	45 (45.9%)	2.42 (1.48,3.94)	1.9 (0.71,5.0)	0.2
ASAIII	10 (47.6%)	11 (52.4%)	1.86 (0.76,4.6)	2.3 (0.46,11.3)	0.3
**Co-existing disease**					
Yes	43 (50.6%)	42 (49.4%)	1.8 (1.1,2.96)	0.848 (0.329,2.19)	0.73
No	92 (36.2%)	162 (63.8%)	1	1	
**Operation Room temperature**					
<26c^o^	122 (49.4%)	125 (50.4%)	5.9 (3.1,11.2)	6 (2.86,13.23)	0.001*
≥26c^o^	13 (14.13%)	79 (85.87%)	1	1	
**Warmed fluid administered**					
Yes	46 (27.9%)	119 (72.1%)	1	1	
No	89 (51.14%)	85 (48.86)	2.7 (1.7,4.25)	2.3 (1.28,4.05)	0.005*
≤500	64 (35.2%)	118 (64.8%)	1	1	
**The volume of Fluid Administer**					
≥500 ml	71 (45.2%)	86 (54.8%)	1.5 (0.98,2.35)	3.6 (1.8,7.2)	0.001*
**Is blood transfused**					
Yes	25 (52.1%)	23 (47.9%)	1	1	
No	110 (37.8%)	181 (62.2%)	0.56 (0.3,1.03)	0.87 (0.38,1.99)	0.73
**Duration of surgery(min)**					
≤120 min	19 (13.8%)	119 (86.2%)	1	1	
>120 min	116 (57.7%)	85 (42.2%)	8.55 (4.89,14.95)	3.37 (1.29,8.79)	0.013*
**Duration of Anesthesia(min)**					
≤120 min	10 (9.1%)	101 (90.9%)	1	1	
>120 min	125 (54.8%)	103 (45.2%)	12.23 (6.09,24.7)	3.79 (1.25,11.5)	0.019*

* = p-value<0.05 1 = reference.

## 4 Discussion

This study reviled that magnitude of intra-operative hypothermia was 39.8% indicating a higher magnitude. This finding is higher than a study done in Kenya 30% [23]. These variations could be due to differences in the technique of measurement and may be due to differences in sample size. While this result was lower than a study done in the United State of America 52% [27] and Uganda 71.7% [28]. This possible reason could be due to variations in clinical setups, population, and seasonal variation.

In this study neonates [AOR = 15; 95%CI=(2.256–101.8)] and infants [AOR = 8.4; 95%CI= (1.548–45.86)] were statistically significant associated with intra-operative hypothermia. This result was consistent with a study done in Malaysia [26]. This might be due to undeveloped physiological thermoregulation mechanism, less subcutaneous tissue, and high body surface area to volume ratio contribute for hypothermia.

Room temperature less than 26c^o^ [AOR; 6, (95%CI, 2.859, 13.231)] was significantly associated with intra-operative hypothermia. This result was similar to a study done in India [8]. Also, Patients who received un-warm fluid [AOR, 2.2, (95%CI, 1.28, 4.05)] during intra-operative were significantly associated with intra-operative hypothermia. This result is consistent with a study conducted in 2011 in the USA that showed, pre-warming administer fluids and other materials reduce intra-operative hypothermia [12].

In this study administration of greater than 1 L of fluid was significantly associated with intra-operative hypothermia. This was similar to a study conducted in Turkey^.^ [1]. The possible reason could be due to redistribution of a high volume of fluid to the system and results reduction of heat.

In this study pediatric patients with a duration of surgery greater than 120 min [AOR; 3.4, (95%CI, 1.297, 8.797)] and duration of anesthesia greater than 120 min [(AOR; 3.8, (95%CI; 1.25, 11.53)] were significantly associated with intra-operative hypothermia. This study was similar to a study conducted USA and Brazile [11,29]. This might be due to suppuration of the thermoregulatory center by anesthetic agents, prolonged heat loss, and increased intravenous fluid administration to replace the fluid. So, surgeons, anesthetists, and nurses should take great responsibility in the reduction of factors causing intraoperative hypothermia in pediatrics patients.

## 5 Conclusion

The magnitude of intra-operative hypothermia in this study was high. So, adherence must be done in the prevention of intraoperative hypothermia by health professionals such as increasing operation room temperature, warming administered fluid, optimal fluid administration, and reduction of the duration of surgery and anesthesia.

## 6 Limitation of study

The limitations of this study were, the first use of a digital axillary thermometer us a measurement of core body temperature may lead to measurement errors, second, blinding was not applied, third a broad range of age group from neonates to adolescents’ might affect the outcome, and fourthly high percentage of infant and toddlers patients might affect the strength of the study.

## Ethics approval and consent

Ethical clearance was obtained from 10.13039/501100007941Addis Ababa University institutional review board and an Official support letter was given from referral hospitals of Addis Ababa as well informed written consent was secured from each study participant's family. Confidentiality was assured throughout the research.

## Funding

Not funded.

## Authors' contributions

All authors contributed to the inception, design, analysis, interpretation, and drafting of the research manuscript. Also, all authors read and approved the revised manuscript for publication.

## Registration of research studies

Name of the registry: http://www.researchregistry.com.

Unique Identifying number or registration ID: researchregistry7388.

Hyperlink to your specific registration (must be publicly accessible and will be checked): https://www.researchregistry.com/browse-the-registry#home/?view_2_sort=field_21|asc.

## Guarantor

Mr. Getachew Mekete.

## Consent for publication

Not applicable.

## Availability of data and material

The data of this study will be available from the corresponding author on reasonable request.

## Competing interest

The authors declare there is no competing interest in this work.

## Provenance and peer review

Not commissioned, externally peer-reviewed.

## Declaration of competing interest

Nothing to declare.

## Acronyms and Abbreviations

AOR: Adjusted Odd Ratio
ASA: American Society of Anesthesiologists
C.I: Confidence interval
COR: Crude odd ratio
BSA: Body Surface Area
ENT: Ear Nose Throat
GA: General Anesthesia
GIT: Gastrointestinal Tracts
PACU: Post Anesthesia Care Unit
SPSS: Statistical Package for Social Sciences
